# Comparación de la microdureza superficial de discos de resina acrílica de termocurado y autocurado en distintos periodos de tiempo

**DOI:** 10.21142/2523-2754-0902-2021-054

**Published:** 2021-06-21

**Authors:** Sarahí Paola Carbajal Córdova, Gustavo Augusto Huertas Mogollón

**Affiliations:** 1 Universidad Científica del Sur. Lima, Perú. paocarcor_3@hotmail.com, gus_tavo_hm@hotmail.com Universidad Científica del Sur Universidad Científica del Sur Lima Peru paocarcor_3@hotmail.com gus_tavo_hm@hotmail.com

**Keywords:** dureza, superficial, acrílico, resina, hardness, surface, acrylic, resin

## Abstract

**Objetivo:**

Comparar la microdureza superficial de discos de acrílico de autocurado y termocurado en distintos periodos de tiempo.

**Metodología:**

Este estudio experimental, un ensayo en laboratorio *in vitro,* incluyó 80 discos de acrílico (Vitalloy), 40 de autocurado (A) y 40 de termocurado (T) divididos en 8 subgrupos: grupo control de autocurado GA0 (n = 10), grupo control de termocurado GT0 (n = 10), grupo de autocurado almancenado 1 día GA1 (n = 10), grupo de termocurado almacenado 1 día GT1 (n = 10), grupo de autocurado almacenado 7 días GA7 (n = 10), grupo de termocurado almacenado 7 días GT7 (n = 10), grupo de autocurado almacenado 21 días GA21 (n = 10), grupo de termocurado almacenado 21 días GT21 (n = 10); luego se evaluó la microdureza superficial Vickers de los discos de resina acrílica previamente almacenados en suero fisiológico en los distintos periodos de tiempo. Posteriormente, los datos fueron analizados estadísticamente mediante test de Anova y Tukey. El nivel de significancia estadística se tomó como p < 0,05.

**Resultados:**

Los valores encontrados fueron en el GT0 (20,45 ± 0,93 VHN); GA0 (16,25 ± 0,79 VHN); GT1 (20,08 ± 0,66 VHN); GA1 (15,97 ± 0,78 VHN); GT7 (19,57 ± 1,54 VHN); GA7 (14,33 ± 0,48 VHN); GT21 (19,17 ± 1,26 VHN); GA21 (14,64 ± 0,52 VHN), presentando diferencias estadísticamente significativas ambos grupos autocurado y termocurado (p < 0,001).

**Conclusión:**

Los grupos de resinas acrílicas de termocurado presentaron mejores valores de microdureza que los grupos de resinas acrílicas de autocurado en los distintos periodos de tiempo evaluados.

## Abstract (en)

**Objective:**

To compare surface microhardness of self-curing and heat-curing acrylic discs in different periods of time.

**Methodology:**

This *in vitro* laboratory study, consisted of 80 acrylic discs (Vitalloy), 40 self-cured (A) and 40 heat-cured (T) divided into 8 subgroups: self-cure control group GA0 (n=10), heat-cure control group GT0 (n=10), self-cure group stored 1 day GA1 (n=10), heat-cure group stored 1 day GT1 (n=10), self-cure group stored 7 days GA7 (n=10), heat-cure group stored 7 days GT7 (n=10), self-cure group stored 21 days GA21 (n=10), and heat-cure group stored 21 days GT21 (n=10). Vickers surface microhardness of the acrylic resin discs previously stored in physiological serum was then evaluated at the different time periods. Subsequently, the data was statistically analyzed using ANOVA and TUKEY tests. The level of statistical significance was set at P <0.05.

**Results:**

The values found were in GT0 (20.45±0.93 Vickers hardness number [VHN]); GA0 (16.25±0.79 VHN); GT1 (20.08±0.66 VHN); GA1 (15.97±0.78 VHN); GT7 (19.57±1.54 VHN); GA7 (14.33±0.48 VHN); GT21 (19.17±1.26 VHN); GA21 (14.64±0.52 VHN), with statistically significant differences between the self-cure and heat-cure groups (P<0.001).

**Conclusion:**

The groups of heat-curing acrylic resins presented better microhardness values than the groups of self-curing acrylic resins in the different time periods evaluated.

## INTRODUCCIÓN

Los provisionales son de suma importancia para el uso clínico, por lo que se debe tomar en cuenta un material que ofrezca las mejores propiedades físicas, mecánicas y biológicas ^1^. Los materiales más utilizados son la resina de polimetil metacrilato (PMMA), la resina de polietil metacrilato (PEMA), la resina de polivinil metacrilato, la resina bis acrílica y los dimetacrilatos de uretano curados por luz ^2^.

El material para un provisional debe satisfacer distintos requerimientos, incluyendo resistencia a la fractura, ajuste marginal, estabilidad del color, resistencia al desgaste, compatibilidad tisular, facilidad de manipulación, buen costo, proporcionar un tiempo de trabajo adecuado y no debe ser tóxico ^3^^,^^4^. 

Se debe tener en cuenta, además, la estética, la comodidad, el habla, la salud periodontal, la oclusión y la estabilidad en el tiempo que requiera el plan de tratamiento ^5^ para identificar un resultado óptimo antes de la finalización del tratamiento ^6^ y evaluar la alteración de la dimensión vertical y la conformación de tejido blando ^5^. Una restauración provisional óptima debe proteger la preparación subyacente, la pulpa ^7^. Las cualidades del PMMA son su biocompatibilidad, confiabilidad, relativa facilidad de manipulación y baja toxicidad.

Las resinas acrílicas de PMMA también sirven para bases de prótesis removibles parciales y totales, debido a su facilidad de procesamiento, bajo costo, peso ligero y capacidad de igualación de colores ^8^^,^^9^. La PMMA de termocurado es activada por el calor, que produce la reacción química entre el monómero y el polímero, lo que da como resultado una polimerización más completa y, por lo tanto, una mayor contracción dimensional, que generan una mayor discrepancia marginal ^10^.

Los compuestos dentales todavía se polimerizan a través del enlace de vinilo de los monómeros de metacrilato. Una desventaja es la conversión incompleta y la sorción/solubilidad en agua, que disminuyen la estabilidad de la matriz polimérica y pueden provocar una vida clínica menos óptima o la degradación de estos compuestos ^11^. Algunos estudios sugieren que esto último proviene de la degradación química de polímeros de metacrilato, debido a la reacción de hidrólisis catalizada por enzimas del enlace éster, lo que produciría un déficit en la resistencia superficial y las propiedades en general ^12^. La humedad también juega un papel importante junto con el efecto de las esterasas salivales que puede reducir la longitud de las cadenas de polímeros, lo que lleva a la fatiga de la resina acrílica ^7^. La reducción de la dureza superficial puede ocurrir debido al fenómeno de sorción de agua por materiales poliméricos ^13^.

Respecto de las propiedades de las resinas acrílicas (PMMA), estas cuentan con poco relleno, por lo que serían más propensas al desgaste superficial; sin embargo, la ausencia de rellenos y la polaridad debido a la naturaleza monofuncional de sus moléculas las hace mas estables en cuanto al color y, a la vez, hace que respondan mejor a las técnicas de pulido tradicionales ^14^. Una superficie lisa puede aumentar la estabilidad del color y absorber mucha agua puede reducirla ^15^. 

La adaptación marginal es de suma importancia en las restauraciones provisionales porque determina si la restauración puede proteger el diente de ataques térmicos, bacterianos y químicos ^10^.

En las resinas acrílicas PMMA, las propiedades físicas dependen del grado de conversión de sus compuestos ^16^; en cuanto a las resinas acrílicas de autocurado, son activadas quimicamente por aminas terciarias, lo que genera un mayor nivel de monómeros residuales y mayor porosidad; las resinas acrílicas de termocurado son activadas por un agente físico para reducir estos monómeros residuales al ser polimerizadas a altas temperaturas ^17^. Se nota la presencia de BPO (peróxido de benzoilo) como iniciador del sistema de resina, el cual se descompone durante el inicio de la polimerización del MMA y proporciona radicales libres para una mayor propagación del polímero, con mayor temperatura habría mayor conversión de del material ^(18, 19)^. Al sumergirlo en agua a 50 grados centígrados, se puede mejorar su dureza superficial y reducir la cantidad de monómeros residuales de MMA en un 80%, así como obtener un aumento significativo en la microdureza ^17^. 

La dureza de un material sólido se puede definir como su resistencia a un cambio de forma permanente cuando se aplica una fuerza de compresión constante ^20^. Se usa como un indicador de densidad y se puede hipotetizar que un material más denso sería más resistente al desgaste y el deterioro de la superficie ^21^. En este estudio, se determinó el VHN (prueba de microdureza), que se basa en la capacidad de la superficie de cualquier material para resistir la penetración de una punta específica con una carga determinada y durante un tiempo específico ^22^. En estudios en los que se analizaba la dureza superficial de materiales sometidos a cargas oclusales, tanto funcionales como parafuncionales, encontraron que las resinas acrílicas tienen mejores valores en dureza superficial ^11^. El término dureza está relacionado con la resistencia del material a la penetración ^13^; por lo tanto, la dureza del material se determina mediante pruebas estandarizadas que promueven la penetración de una punta en este material con el uso de un instrumento específico conocido como durómetro ^23^. 

Actualmente, todavía existe una gran incertidumbre en torno a qué tipo de material provisional se debe usar ^17^. No hay un consenso que evidencie estudios de microdureza de resinas acrílicas comparadas en el tiempo, sobre todo porque es un material cotidianamente utilizado. Por tanto, el propósito del presente estudio es comparar la microdureza superficial de discos de acrílico de termocurado y autocurado en distintos periodos de tiempo.

## MATERIALES Y MÉTODOS

Este estudio fue aprobado por el Comité de Ética de la Universidad Científica del Sur (CIEI-Cientifica) y exonerado por no presentar vulnerabilidad ética con código de registro n.º 149-2020-PRO99. 

La muestra consistió en 80 discos de resina acrílica PMMA de la marca Vitalloy (Vitalloy, Tarrillo Barba, Lima, Perú), distribuidos en ocho subgrupos: grupo control de autocurado GA0 (n = 10), grupo control de termocurado GT0 (n = 10), grupo autocurado almacenado 1 día en suero fisiológico GA1 (n = 10), grupo termocurado almacenado 1 día en suero fisiológico GT1 (n = 10), grupo autocurado almacenado 7 días en suero fisiológico GA7 (n = 10), grupo termocurado almacenado 7 días es suero fisiológico GT7 (n = 10), grupo autocurado almacenado 21 días en suero fisiológico GA21 (n = 10), grupo termocurado almacenado 21 días en suero fisiológico GT21 (n = 10) al 9%. El tamaño de muestra ha sido determinado mediante una fórmula de tamaño muestral para comparar dos promedios (el promedio de microdureza en todos grupos) consignando los siguientes datos según el articulo base: nivel de confianza = 99%, poder de la prueba = 95%, media 1= 18,00, media 2 = 15,00, desviación estándar del grupo control = 1, total por grupo n = 4 ^12^; pero se decidió trabajar con 10 discos tomando como referencia distintos trabajos de investigación ^19^. 

Los criterios de selección estuvieron basados en incluir discos de resina acrílica de termocurado de la marca Vitalloy, con medidas de 13 mm de diámetro por 4 mm de espesor, con las mismas medidas para los discos de resina acrílica de autocurado. Asimismo, se tomó en cuenta que todos los discos tuvieran la superficie lisa y se excluyó aquellos que presentaban burbujas, fracturas o fisuras y superficie rugosa, tanto si eran de termocurado o de autocurado.

Para la fabricación de los discos de resina acrílica de termocurado, se utilizó polvo de acrílico de termocurado color 59 (Vitalloy, Tarrillo Barba, Lima, Perú) y líquido (Vitalloy, Tarrillo Barba, Lima, Perú), con las siguientes proporciones: 1:2 líquido y polvo, respectivamente, 6 ml de líquido por 12 g de polvo para 5 discos. Para el molde de los discos se fabricaron anillos de aluminio de 14 mm de diámetro por 5 mm de espesor, los cuales se realizaron por medio de goteo de cera base rosada calentada. Al obtener los discos de cera, estos fueron sumergidos en yeso tipo III (Pentadur, Penta Industrias S.A.C., Lima, Perú), que contenía una mufla, para que seguidmente engrane la contramufla y se ajuste lo mejor posible. Así se pudo continuar vertiendo yeso piedra tipo III y esperar a que este fragüe para luego retirar la contramufla y sacar los discos de cera en el yeso ya fraguado. Antes de colocar la mezcla de polvo líquido sobre la mufla, se aplicó aislante para acrílico (Vitalloy, Tarrillo Barba, Lima, Perú).

La mezcla del polvo y líquido consistió en verter primero el líquido y luego el polvo en un recipiente de vidrio, y homogenizar la mezcla con una espátula hasta llegar a la fase arenosa. Seguidamente, se cubrió con una bolsa plástica transparente para que su polimerización no se viera afectada por el ambiente; en esta etapa fue cuando se manipuló y se llevó a la mufla por medio de un plástico previamente lavado, para así no contaminar la mezcla. Al tener llenos los espacios con resina acrílica, se engranó con la contramufla y se aplicaron 2 prensados. Cuando se retiró la contramufla, se eliminaron los excesos con un bisturí de hoja #15 y se volvió a colocar la contramufla para llevarla a una olla con agua a 70 grados centígrados por 1 hora y media; luego, se aumentó la temperatura a 100 grados centígrados por un lapso de media hora más ^24^. 

Al sacar los discos de acrílico de la mufla, se llevaron al proceso de pulido, que constó primero en retirar excesos con una fresa de carburo de doble corte; luego, con una piedra rosada cilíndrica se eliminaron irregularidades y con un caucho verde para acrílico se terminó de eliminar las irregularidades más pequeñas. Se continuó llevando los discos a una máquina de pulido con felpas; con la primera se aplicaba piedra pómez para pulido y con la segunda, *rush* para sacar brillo. Por último, se corroboraron los criterios de inclusión.

Para la confección de los discos de resina acrílica de autocurado se utilizaron anillos de 14 mm de diámetro y 5 mm de espesor. La mezcla para la resina acrílica de autocurado se tomó como proporción 5:3 polvo y líquido, respectivamente, utilizando un total de 72 gramos de polvo color 59 (Vitalloy, Tarrillo Barba, Lima, Perú) y 48 mililitros de líquido (Vitalloy, Tarrillo Barba, Lima, Perú).

Se realizó la mezcla sobre un recipiente de vidrio, al igual que con los discos de termocurado, para luego colocarla dentro de los anillos de aluminio que se encontraban sobre una platina de vidrio con vaselina. El otro extremo fue cubierto con una lámina portaobjetos y se ejerció presión para que los excesos escurran. Luego de 5 minutos, los discos se encontraban polimerizados y se procedió a realizar el pulido, para así conseguir una superficie lisa y brillante.

**Figura 1:**
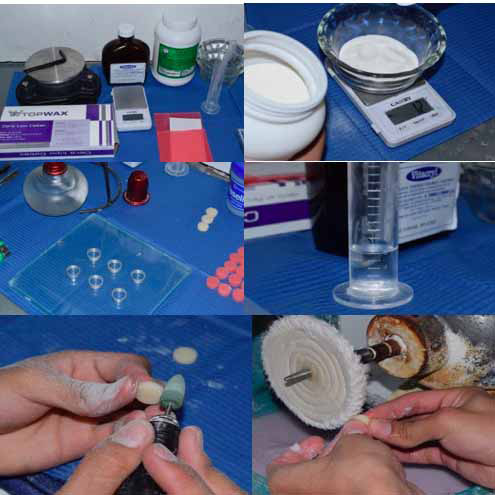
Procedimiento de confeccion de discos de resina acrilica y pulido.

Luego de obtener los 80 discos de resina acrílica de termocurado y autocurado, estos fueron divididos de manera aleatoria en 8 grupos ya mencionados anteriormente. Los contenedores de cada grupo fueron de plástico, sellados y etiquetados según el grupo que contenía (Figura 1).

**Figura 2:**
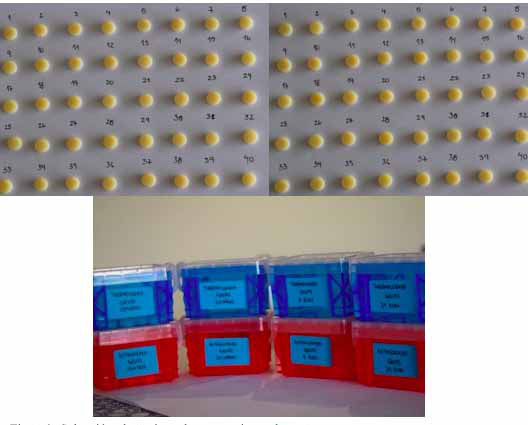
Discos de resina acrílica de termocurado y de autocurado almacenados de manera aleatoria.

La solución que se utilizó para el almacenamiento de los grupos GA1, GT1, GA7, GT7, GA21 y GT21 fue suero fisiológico al 9%; esta solución tenía que cubrir los discos. Los grupos GA1 y GT1 estuvieron 1 dia sumergidos en suero fisiologico a 37 oC, aproximadamente, antes de la prueba de microdureza Vickers. Los grupos GA7 y GT7 estuvieron sumergidos por 7 días en suero a 37 oC, antes de la prueba de microdureza, y los grupos GA21 y GT21 estuvieron 21 días sumergidos en suero a 37 oC antes de la prueba de microdureza (Figura 2).

**Figura 3:**
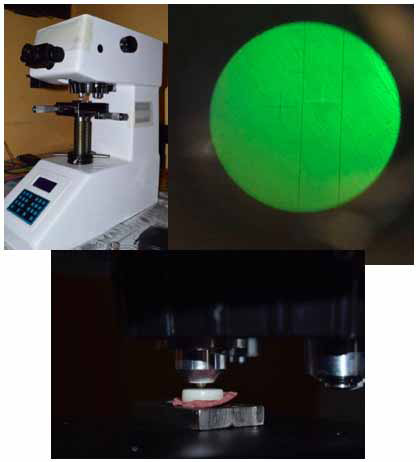
Prueba de microdureza Vickers superficial en microdurómetro.

La prueba de microdureza Vickers se realizó en el laboratorio High Technology Laboratory Certificate, con un microdurómetro Vickers CV400AAT, marca LG (torreta motorizada con microscopio de medición analógico, control de carga automática, con dos recorridos ópticos y sistema de cálculo de dureza integrado). Cada disco fue sometido a dos indentaciones, con una carga de 50 gramos durante 10 segundos cada una, para luego sacar un promedio entre las dos indentaciones por disco. La fórmula nos indica que VHN se expresa en MPa, si P es la carga aplicada está en N y d es la diagonal de la sangría en mm. ATAC representa la verdadera área de contacto ^24^ (Figura 3).

El análisis estadístico se realizó con la ayuda del programa SPSS Statistics V24. Este procedimiento comenzó con la estadística descriptiva, que consistió en la presentación de las medidas de tendencia central (media y mediana) y las medidas de dispersión (desviación estándar, mínimo, máximo, rango y varianza) de la variable microdureza en cada grupo de estudio. A continuación, se evaluó la normalidad en los grupos de estudio mediante la prueba de Shapiro-Wilk. Finalmente, se realizó las comparaciones entre grupos mediante el test de Anova de un factor y la prueba de comparaciones múltiples de Tukey, todo se trabajó con un nivel de significancia de p < 0,05.

## RESULTADOS

Luego de realizar el ensayo de microdureza Vickers (VHN) superficial en discos de resina acrílica, se encontraron diversos valores para cada grupo de acrílicos de autocurado y termocurado (Anexos 2, 3, 4 y 5). Los resultados promedio de las indentaciones se muestran en la Tabla 1 en valores de VHN. Para el GT0, el valor promedio para la variable microdureza fue 20,45 ± 0,93 VHN; el GA0 arrojó un valor promedio de 16,25 ± 0,79 VHN; para el GT1 dio un valor promedio de 20,08 ± 0,66 VHN; en el GA1 mostró un valor promedio de 15,97 ± 0,78 VHN; el GT7 dio un valor promedio de 19,57 ± 1,54 VHN; el GA7 arrojó un valor promedio de 14,33 ± 0,48 VHN; el valor promedio de GT21 fue de 19,17 ± 1,26 VHN y, finalmente, el valor promedio de GA21 fue de 14,64 ± 0,52 VHN.

**Tabla 1:** Evaluación de la microdureza Vickers superficial de resinas acrílicas de termocurado y autocurado

TIEMPO	MODO	Microdureza Vickers superficial de resinas acrílicas de termocurado y autocurado						
		n	Media	D. E.	Mín.	Máx.	Varianza	P
Control	T	10	20,45	0,93	19,2	21,7	0,87	<0,001
	A	10	16,25	0,79	15,1	17,5	0,64	
1 día	T	10	20,08	0,66	18,9	21,2	0,44	
	A	10	15,97	0,78	15,1	17,7	0,62	
7 días	T	10	19,57	1,54	16,7	21,9	2,38	
	A	10	14,33	0,48	13,7	15,2	0,23	
21 días	T	10	19,17	1,26	16,8	20,7	1,59	
	A	10	14,64	0,52	13,7	15,5	0,27	

*T: Termocurado *D. E.: Desviación estándar

*A: Autocurado *P: Test de Anova de un factor

Por su parte, el valor máximo para GT0 fue de 21,7 VHN y el valor mínimo fue 19,2 VHN; para GA0 el valor máximo fue de 17,5 VHN y el valor mínimo fue de 15,1 VHN; el GT1 mostró como valor máximo 21,2 HVN y valor mínimo 18,9 HVN; para el GA1 el valor máximo fue de 17,7 HVN y valor mínimo 15,1 HVN; en el GT7 el valor máximo fue 21,9 HVN y el valor mínimo de 16,7 HVN; mientras que para el GA7 el valor máximo fue 15,2 HVN y valor mínimo 13,7 HVN; para el GT21 el valor máximo fue 20,7 HVN y valor mino 16,8 HVN, por último el GA21 mostró un valor máximo de 15,5 HVN y valor mínimo de 13,7 HVN.

**Figura 4:**
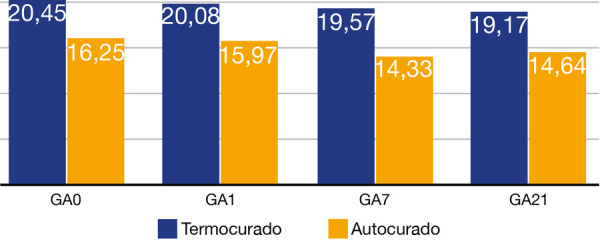
Resultados de la evaluación de la microdureza Vickers superficial de resinas acrílicas de termocurado y autocurado.

Se encontraron diferencias estadísticamente significativas (p < 0,05) entre todos los grupos estudiados (p < 0,001) (Figura 4).

**Figura 5:**
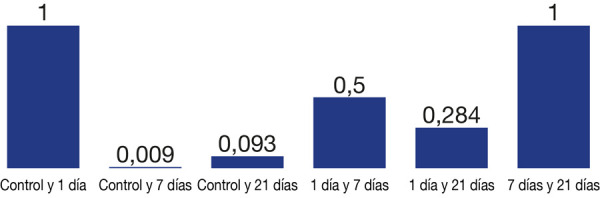
Resultado de las comparaciones múltiples de Tukey de los grupos evaluados de autocurado en los distintos periodos de tiempo (valores HVR).

Se realizó el test de comparaciones múltiples de Tukey (Tabla 2), en el que se encontraron diferencias estadísticamente significativas (p < 0,05) entre GT0 y GT0 (p < 0,001); se encontró que en GT0 y GA1 (p < 0,001) hubo diferencia estadísticamente significativa; también se encontró diferencias estadísticamente significativas entre GT0 y GA7 (p < 0,001); entre GT0 y GA21 (p < 0,001) también se encontraron diferencias estadísticamente significativas; hubo diferencia estadísticamente significativa entre GA0 y GT1 (p < 0,001), al igual que entre GA0 y GT7 (p < 0,001) diferencia estadísticamente significativa; entre GA0 y GA7 (p < 0,009) también hubo diferencia estadísticamente significativa; al igual entre GA0 y GT21 (p < <0,001); se encontró también diferencia estadísticamente significativa entre GT1 y GA1 (p < 0,001); en GT1 y GA7 (p < 0,001); también se encontró diferencia estadísticamente significativa; hubo diferencia estadísticamente significativa entre GT1 y GA21 (p < 0,001); entre GA1 y GT7 (p < 0,001) hubo diferencia significativa; entre GA1 y GT21 (p < 0,001) también hubo diferencia estadísticamente significativa; GT7 y GA7 (p < 0,001) hay diferencia estadísticamente significativa; se encontró diferencia estadísticamente significativa entre GT7 y GA21 (p < 0,001); al igual que GA7 y GT21 (p < 0,001) que se encontró diferencia estadísticamente significativa; por último, se encontró diferencia estadísticamente significativa entre GT21 y GA21 (Figuras 5 y 6).

**Tabla 2:** Comparaciones múltiples de Tukey de los grupos evaluados en los distintos periodos de tiempos

GRUPO	GRUPO	P	95% de intervalo deconfianza	
			Límite inferior	Límite superior
Control termocurado	Control autocurado	<0,001	2,56	5,84
	1d termocurado	0,998	-1,27	2
	1d autocurado	<0,001	2,84	6,12
	7d termocurado	0,740	-0,76	2,52
	7d autocurado	<0,001	4,48	7,76
	21d termocurado	0,260	-0,36	2,92
	21d autocurado	<0,001	4,07	7,55
Control autocurado	1d termocurado	<0,001	-5,47	-2,19
	1d autocurado	1,000	-1,36	1,92
	7d termocurado	<0,001	-4,96	-1,68
	7d autocurado	0,009	0,28	3,56
	21d termocurado	<0,001	-4,56	-1,28
	21d autocurado	0,093	-0,13	3,35
1d termocurado	1d autocurado	<0,001	2,47	5,75
	7d termocurado	0,983	-1,13	2,15
	7d autocurado	<0,001	4,11	7,39
	21d termocurado	0,705	-0,73	2,55
	21d autocurado	<0,001	3,7	7,18
1d autocurado	7d termocurado	<0,001	-5,24	-1,96
	7d autocurado	0,500	<0,0011	3,28
	21d termocurado	<0,001	-4,84	-1,56
	21d autocurado	0,284	-0,41	3,07
7d termocurado	7d autocurado	<0,001	3,6	6,88
	21d termocurado	0,996	-1,24	2,04
	21d autocurado	<0,001	3,19	6,67
7d autocurado	21d termocurado	<0,001	-6,48	-3,2
	21d autocurado	1,000	-2,05	1,43
21d termocurado	21d autocurado	<0,001	2,79	6,26

*P: test de comparaciones múltiples de Tukey

**Figura 6:**
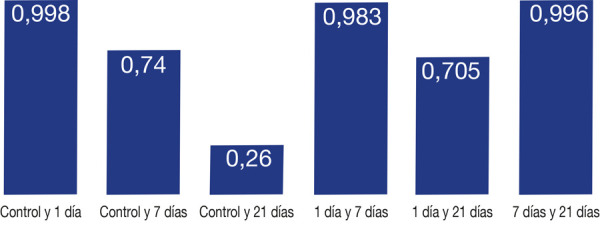
Resultado de las comparaciones múltiples de Tukey de los grupos evaluados de termocurado en los distintos periodos de tiempo (valores HVR).

## DISCUSIÓN

Se han realizado diversos estudios con respecto a materiales de restauración provisional, con el fin de analizar sus propiedades físicas y así decidir por el material más adecuado de acuerdo con el plan de tratamiento. 

Los datos que resultaron muestran diferentes valores de microdureza superficial entre todos los grupos analizados, lo que evidencia una disminución de microdureza superficial con respecto a los valores de los grupos control de acrílicos de autocurado y termocurado, en estudios que anteceden a este ^25^^-^^28^, la reducción de la microdureza superficial puede ocurrir debido al fenómeno de sorción de agua por materiales poliméricos, por lo que el exceso de agua puede causar una desunión de la matriz de relleno ^29^. Quizás sea por este fenómeno que la microdureza superficial de los discos de resina acrílica de PMMA haya ido disminuyendo con el pasar de los días respecto de las muestras de los grupos control.

Un factor importante para tener en cuenta entre los acrílicos de autocurado y termocurado es que existen diferencias en cuanto a sus propiedades de microdureza y resistencia a la abrasión, lo que se debe a los radicales libres que pueden quedar inactivos en los acrílicos de autocurado. Asimismo, se constató que el proceso térmico de las resinas acrílicas de termocurado, es el factor dominante para mejorar la microdureza superficial de las resinas acrílicas ^17^. Por eso se observan diferencias estadísticamente significativas entre los grupos de autocurado y termocurado debido al factor físico por la que son activadas; en este caso, las resinas acrílicas de termocurado, polimerizadas a altas temperaturas son las que presentan valores más altos de dureza superficial.

Un estudio sobre materiales para prótesis removibles, con muestras de resina acrílica de autocurado y termocurado, sugiere utilizar resinas acrílicas de termocurado para prótesis removibles, de acuerdo con los valores encontrados de microdureza superficial que estaría relacionado con la presencia de monómero residual, considerando que a mayor cantidad de monómeros residuales menor será la microdureza superficial ^30^^,^^22^.

Otro factor que puede haber influido en la reducción de la dureza de las resinas acrílicas es la presencia de monómero residual, que afecta negativamente a las propiedades mecánicas debido al efecto plastificante que disminuye las fuerzas entre cadenas y permite que la deformación se produzca fácilmente bajo carga ^26^.

La adaptación marginal de coronas de autocurado mencionada en un estudio refiere que existe una disminución de la adaptación marginal por la contracción de la polimerización, la reacción de polimerizacion exotérmica y la irritación asociada con el monómero, lo que deja niveles relativamente bajos de acabado y adaptación marginal ^31^.

Quizás sea esta otra de las razones por las cuales los discos de resina acrílica de autocurado presentan valores inferiores de dureza que los discos de resina acrílica de termocurado.

En un estudio previo donde analizaron la microdureza de muestras de acrílico de autocurado a temperatura ambiente almacenadas 90 días y en inmersión de agua, se halló que, en las pruebas a temperatura ambiente, la dureza aumentó de 23,2 VHN a 27,1 VHN con el tiempo, mientras que en las muestras en inmersión en agua la dureza disminuyó de 25 VHN a 16 VHN, debido a su ablandamiento. Cuando las resinas acrílicas se sumergen en agua, los monómeros residuales se liberan y la absorción de agua ocurre simultáneamente. Estos procesos son controlados por difusión y dependen del tiempo. Se ha demostrado que tanto el agua como el monómero residual actúan como plastificantes, lo que afecta la resistencia de las resinas polimerizadas ^27^.

Un estudio en el que se sometió a las muestras a sustancias que simulaban alimentos demostró que el mayor cambio en la dureza de los compuestos de resina se presentó después del acondicionamiento con estas soluciones en los primeros 7 días, debido a que las soluciones a las que fueron expuestas las muestras pueden quedar atrapadas alrededor de los márgenes y en porosidades del material provisional ^32^. Quizás sea esta la razón por la que se presentó diferencia significativa en los discos de resina acrílica de autocurado almacenados 7 días.

Otro estudio evaluó la microdureza Knoop de las resinas acrílicas de prótesis removibles polimerizadas convencionalmente y obtuvieron valores medios en el rango de 16,9-19,66 KHN28.

Este estudio se evaluó la microdureza Vickers y se efectuó la comparación con las resinas acrílicas de autocurado. Los valores medios fueron entre 14,64-16,25 HVN. El análisis de microdureza Vickers revela que, a mayor carga, mayores serán los valores de microdureza (HV), mientras que en el análisis Knoop es inverso, a menor carga mayores serán los valores de microdureza (HK)^33^. Esto nos llevaría a no realizar una comparación entre estudios de microdureza Vickers y Knoop porque son pruebas distintas.

## CONCLUSIONES

Se concluye que los discos de resina acrílica de autocurado, el grupo que presentó mayor valor de microdureza superficial fue el grupo control (GA0), mientras que el grupo que presentó el menor valor de microdureza fue el grupo de discos de resina acrílica almacenados 7 días (GA7), por lo tanto, solo se evidenció diferencia significativa entre el grupo control GA0 y el grupo de discos almacenados 7 días GA7 (p < 0,009), mientras que en los discos de resina acrílica de termocurado el grupo que presentó mayor valor de microdureza superficial fue el grupo control (GT0), y el grupo con menor valor fue el de discos de acrílico almacenados 21 días (GT21), lo que no evidencia diferencia significativa entre los grupos de discos de resina acrílica de termocurado.

## Fuente de financiamiento:

Autofinanciado
